# Acteoside Suppresses RANKL-Mediated Osteoclastogenesis by Inhibiting c-Fos Induction and NF-κB Pathway and Attenuating ROS Production

**DOI:** 10.1371/journal.pone.0080873

**Published:** 2013-12-04

**Authors:** Seung-Youp Lee, Keun-Soo Lee, Sea Hyun Yi, Sung-Ho Kook, Jeong-Chae Lee

**Affiliations:** 1 Research Institute of Clinical Medicine of Chonbuk National University, Biomedical Research Institute of Chonbuk National University Hospital, Jeonju, Chonbuk, South Korea; 2 Department of Orthodontics, Institute of Oral Biosciences and School of Dentistry, Chonbuk National University, Jeonju, Chonbuk, South Korea; 3 Department of Bioactive Material Sciences, Research Center of Bioactive Materials, Chonbuk National University, Jeonju, Chonbuk, South Korea; National Cancer Institute, United States of America

## Abstract

Numerous studies have reported that inflammatory cytokines are important mediators for osteoclastogenesis, thereby causing excessive bone resorption and osteoporosis. Acteoside, the main active compound of *Rehmannia glutinosa*, which is used widely in traditional Oriental medicine, has anti-inflammatory and antioxidant potentials. In this study, we found that acteoside markedly inhibited osteoclast differentiation and formation from bone marrow macrophages (BMMs) and RAW264.7 macrophages stimulated by the receptor activator of nuclear factor-kappaB (NF-κB) ligand (RANKL). Acteoside pretreatment also prevented bone resorption by mature osteoclasts in a dose-dependent manner. Acteoside (10 µM) attenuated RANKL-stimulated activation of p38 kinase, extracellular signal-regulated kinases, and c-Jun N-terminal kinase, and also suppressed NF-κB activation by inhibiting phosphorylation of the p65 subunit and the inhibitor κBα. In addition, RANKL-mediated increases in the expression of c-Fos and nuclear factor of activated T-cells, cytoplasmic 1 (NFATc1) and in the production of tumor necrosis factor-α, interleukin (IL)-1β, and IL-6 were apparently inhibited by acteoside pretreatment. Further, oral acteoside reduced ovariectomy-induced bone loss and inflammatory cytokine production to control levels. Our data suggest that acteoside inhibits osteoclast differentiation and maturation from osteoclastic precursors by suppressing RANKL-induced activation of mitogen-activated protein kinases and transcription factors such as NF-κB, c-Fos, and NFATc1. Collectively, these results suggest that acteoside may act as an anti-resorptive agent to reduce bone loss by blocking osteoclast activation.

## Introduction

Bone is constant remodeled by balanced osteoclast and osteoblast activity [1]. Osteoclasts arise from hematopoietic precursor cells of the monocyte/macrophage lineage, while osteoblasts are of the mesenchymal lineage [2]. Abnormal osteoclast activation or reduced osteoblastogenesis can disrupt bone homeostasis, eventually causing diseases such as osteoporosis, arthritis, and bone cancer [3], [4]. Osteoporosis is a common bone disease that leads to an increased risk of fracture. The most common form of osteoporosis is caused by estrogen deficiency in menopausal females. Medications such as corticosteroids and anti-epileptics may also cause an imbalance between bone resorption and formation, which can result in osteoporosis [5].

Many anti-resorptive inhibitors including bisphosphonates, calcitonin, estrogen, and selective estrogen receptor modulators have been used to treat osteoporosis. These inhibitors maintain bone mass by inhibiting osteoclast function [6]. Estrogen replacement therapy is the most popular treatment to prevent and treat post-menopausal osteoporosis. However, long-term estrogen replacement therapy can increase the risk of endometrial and breast cancers. Therefore, many investigators have focused their efforts on developing a new anti-resorptive agent that does not have side effects [7]–[10]. Because osteoclasts function in bone resorption, specifically inhibiting osteoclasts has been considered the main target in numerous studies.

Osteoclasts are multinucleated giant cells formed by mononuclear progenitors of the monocyte/macrophage family via the sequential proliferation, differentiation, and fusion of hematopoietic precursor cells [11]. Macrophage colony stimulating factor (M-CSF) and receptor activator of nuclear factor (NF)-κB (RANK) ligand (RANKL) are essential factors for osteoclast differentiation [12]. In addition, several inflammatory cytokines, such as tumor necrosis factor (TNF) and interleukin (IL)-1β, contribute to osteoclastogenesis by modulating the induction of RANKL, osteoprotegerin, and M-CSF [7], [13]. RANKL binding to the cell surface RANK receptor, results in RANKL/RANK/TNFR associated factors (TRAF) complexes that sequentially activate NF-κB and mitogen-activated protein kinases (MAPKs), including c-Jun N-terminal kinase (JNK), p38 kinase, and extracellular signal-related kinase (ERK) [14]. This activation plays a key role in mediating the osteoclast differentiation, activation, and survival. RANKL also activates the expression of transcription factors such as nuclear factor of activated T-cells, cytoplasmic 1 (NFATc1) and c-Fos, which are essential for osteoclast development [7], [9]. Therefore, RANKL signaling is considered as the main target of anti-resorptive agents that suppress osteoclast activation and bone loss.

Acteoside is the main active compound of *Rehmannia glutinosa*, which is used widely in traditional Oriental medicine [15], [16]. Acteoside is a strong antioxidant and has anti-hepatotoxic, anti-inflammatory, and anti-nociceptive activities [17]–[20]. We previously found that acteoside decreases tyrosinase activity and melanin biosynthesis by regulating ERK signaling [21], protects against reactive oxygen species (ROS)-mediated gingival damage [22], and suppresses mycotoxin-mediated cell damage [23]. These effects are closely related to acteoside's ability to remove ROS and regulate MAPK-mediated signaling. In particular, ROS are suggested to mediator RANKL-induced signaling pathways and cellular events in osteoclasts. Pretreatment with antioxidants inhibited RANKL-induced activation of NF-κB, ERK, and IκBα, thereby suppressing osteoclastogenesis [24]. These findings strongly suggested that, in addition to anti-inflammatory activity, an antioxidant activity is crucial for an anti-resorptive agent, thus acteoside can suppress RANKL-induced osteoclastogenesis.

In the present study, we explored whether acteoside has a therapeutic effect on bone loss. We examined the effects of acteoside on osteoclast differentiation and bone resorption and the related cellular mechanisms using *in vitro* and *in vivo* experimental systems.

## Materials and Methods

### Ethics Statement

Animal care and use practices were approved by the Chonbuk National University Committee on Ethics in the Care and Use of Laboratory Animals (Permit No. CBU 2010-0007). All experiments in this study were carried out according to the guidelines of the Animal Care and Use Committee of the University.

### Mice, Chemicals, and Laboratory Wares

Four-week old female ICR mice were purchased from Orient Bio Inc. (Seoul, Korea) and housed at 22±1°C and 55±5% humidity on a 12 h light/dark cycle with free access to food and water. Acteoside (3,4-dihydroxy-β-phenethyl-*O*-α-rhamnopyranosyl-(1→3)-4-*O*-caffeoyl-β-D-glucopyranoside; C_29_H_36_O_15_) (Fig. S1) was isolated from the leaves of *Rehmannia glutinosa*. Acteoside was dissolved in phosphate-buffered saline (PBS) before use. RANKL, TNF-α, IL-1β, IL-6, and M-CSF were purchased from R & D Systems (Minneapolis, MN, USA). Antibodies specific to c-Fos, p65, p-p65, p-ERK, ERK, JNK, p-JNK, NFATc1, IκBα, p-IκBα, and β-actin were obtained from Santa Cruz Biotechnology (Santa Cruz, CA, USA). Antibodies for p-p38 and p38 were purchased from Cell Signaling Technology (Denvers, MA, USA). Calcium Assay and Osteocalcin (OC) EIA Kits were purchased from BioAssay Systems (Hayward, CA, USA) and Biomedical Technologies (Stoughton, MA, USA), respectively, to determine serum biochemical parameters. Mouse tartrate-resistant acid phosphatase (TRAP) Assay kit (Immunodiagnostic Systems, Scottsdale, AZ, USA) was also used to measure serum TRAP5b level. Unless otherwise specified, additional chemicals were obtained from Sigma Chemical Co. (St. Louis, MO, USA), and laboratory wares were from SPL Life Sciences (Pochun, South Korea).

### Cell Cultures

Bone marrow cells were obtained from the tibiae and femora of 6 week-old female ICR mice according to methods described previously [25]. The bone marrow suspension was incubated in a 100-mm culture dish in the presence of 50 ng/ml M-CSF. After 3 days, adherent cells were used as bone marrow macrophages (BMMs) to induce osteoclastic differentiation. Some bone marrow cells were also incubated for 48 h without M-CSF, and adherent cells were cultured in osteoblast differentiating medium, as described elsewhere [25]. RAW264.7 macrophage cells were cultured in Dulbecco's modified Eagle's medium (DMEM) supplemented with 10% fetal bovine serum (FBS), 2 mM L-glutamine, and antibiotics. These cells were used as a counterpart cell line for BMMs to explore the effect of acteoside on osteoclastogenesis.

### Osteoclastic Differentiation and TRAP Staining

BMMs were pretreated with various concentrations (0–50 µM) of acteoside for 2 h before stimulating with 100 ng/ml RANKL. Culture media was replaced with fresh media on days 2 and 5. After 7 days of incubation, the cultures were fixed in 4% PBS-buffered para-formaldehyde and stained with TRAP using a Sigma Aldrich kit according to the manufacturer's instructions. TRAP-positive cells were counted using optic microscopy, and cells containing 3 or more nuclei were considered to be osteoclasts. RAW 264.7 cells were also exposed to 100 ng/ml RANKL after pretreatment with acteoside for 2 h, and after 7 days of incubation, the cells were processed for TRAP staining.

### Measurement of Cell Viability

Cell viability was determined using water-soluble tetrazolium salt (WST)-8 reagent. In brief, BMMs or RAW264.7 cells cultured in a growth medium containing 10% FBS and antibiotics were treated with 10 µM acteoside or a phenolic compound such as quercetin, luteolin, apigenin, or epigallocatechin-3-gallate (EGCG). WST-8 reagent was added into the cultures after 48 h of incubation. After incubating for an additional 4 h, the WST-8-specific absorbance was measured at 450 nm using a microplate reader (Packard Instrument Co., Downers Grove, IL, USA).

### Bone Resorption Assay

BMMs (1×10^5^ cells/ml) were suspended in α-MEM containing 50 ng/ml M-CSF and 100 ng/ml RANKL, then divided across a 24-well plate coated with calcium-phosphate nano crystals (OAAS-24; Osteoclast Activity Assay Substrate, Oscotec Inc., Choongnam, South Korea) at a density of 2×10^4^ cells/cm^2^ with and without acteoside. After incubating for 7 days, the cells were removed from the plates with 5% sodium hypochlorite, and pit formation was observed under an optic microscope. The resorbed area was also measured by image analyzer and expressed as percentage of the control value.

### Western Blot Analysis

Whole protein lysates were prepared in a lysis buffer as described elsewhere [26]. Cytosolic and nuclear proteins were prepared as described previously [25]. Equal amounts of protein extract were separated by 12–15% SDS-PAGE and blotted onto poly vinyl difluoride membranes. The blots were probed with primary antibodies overnight at 4°C before incubation with secondary antibody in blocking buffer for 1 h. The blots were developed with enhanced chemiluminescence (Amersham Pharmacia Biotech Inc., Buckinghamshire, UK) and exposed on X-ray film (Eastman-Kodak Co., Rochester, NY, USA).

### MAPK Activity Assay

Cells were pretreated with acteoside for 2 h and then stimulated with RANKL for an additional-30 min. MAPK activities were determined using immunometric assay kits, such as the p-p38 kinase assay kit (Assay Designs, Inc., MI, USA), p-ERK enzyme assay kit (Assay Designs), and p-SAPK/JNK sandwich ELISA kit (Cell Signaling Technology, MA, USA). All procedures followed the manufacturer's instructions, and absorbance was measured by a microplate reader.

### Electrophoretic Mobility Shift Assay (EMSA)

DNA-protein binding reactions were performed for 30 min at room temperature, with 10–15 µg protein in 20 µl buffer containing 1 µg/ml BSA, 0.5 µg/µl poly (dI-dC), 5% glycerol, 1 mM DTT, 1 mM PMSF, 10 mM Tris-Cl (pH 7.5), 50 mM NaCl, 30,000 cpm of [α-^32^P] dCTP-labeled oligonucleotides, and the Klenow fragment of DNA polymerase. The samples were separated on 6% polyacrylamide gels, which were dried and exposed to X-ray film (Eastman Kodak Co.) for 12–24 h at −70°C. The oligonucleotide primer sequences specific for NF-κB were 5′-AAG GCC TGT GCT CCG GGA CTT TCC CTG GCC TGG A-3′ and 3′-GGA CAC GAG GCC CTG AAA GGG ACC GGA CCT GGA A-5′.

### NF-κB Luciferase Assay

RAW264.7 macrophages in 24-well plates were transfected with 0.8 µg κB-luciferase reporter vector using 2 µl of Lipofectamine 2000 (Invitrogen, Carlsbad, CA, USA) according to the manufacturer's instructions. At 24 h after transfection, the cells were stimulated with RANKL in the presence and absence of acteoside for 24 h. Cells were resuspended in 100 µl reporter lysis buffer (Promega, Madison, WI, USA). Equal amounts of protein samples were placed into 96-well microplates and mixed with luciferase substrate. Luminescence was measured by using a microplate luminometer (MicroLumat Plus LB 964, Berthold Technologies, Bad Wildbad, Germany). In this experiment, a permeable NF-κB inhibitor peptide (BIOMOL, Butler Pike, PA, USA) was used as positive κB inhibitor.

### Measurement of Cytokines

BMMs or RAW264.7 cells were stimulated with RANKL in the presence of acteoside in 24-well culture plates. After 48 h of incubation, culture supernatants were collected and assessed by ELISA using TNF-α-, IL-1β- and IL-6-specific OptEIA™ kits according to the manufacturer's instructions.

### Real-Time Reverse Transcription-Polymerase Chain Reaction (RT-PCR)

The mRNA expression of osteoclastic markers, such as c-Fos, NFATc1, and TNF-α, was determined by real-time RT-PCR. In brief, total RNA was extracted from macrophages with Trizol reagent according to the manufacturer's instructions (Invitrogen). cDNA was synthesized with 1 µg of total RNA using SuperScript Reverse Transcripatase II and primers (Invitrogen). Power SYBR Green PCR Master Mix (Applied Biosystems, Foster City, CA, USA) was used to detect the accumulation of PCR product during cycling with the ABI 7500 sequence detection system (Applied Biosystems). After denaturation at 95°C for 10 min, PCR was performed using forty 3-step cycles of denaturation at 95°C for 15 sec, annealing at 60°C for 1 sec, and extension at 72°C for 30 sec. All PCR reactions were performed at least in triplicate, and the expression levels were normalized to the housekeeping gene HPRT in the same reaction. This study used the same primer sequences described elsewhere [7].

### Measurement of Intracellular ROS

A stock solution of 2′,7′-dichlorodihydrofluorescein-diacetate (DCFH-DA) (50 mM; Calbiochem, Darmstadt, Germany) was prepared in DMSO and stored at −20°C in the dark. In brief, BMMs (10^6^ cells/ml in 6-well plates) were cultured with 50 ng/ml M-CSF for 24 h and then treated with various concentrations (0–10 µM) of acteoside 2 h before stimulation with 100 ng/ml RANKL. After 1 h of co-incubation, these cells were subsequently incubated with 25 µM DCFH-DA for 30 min. The green fluorescence of 2′,7′-dichlorofluorescein (DCF) was recorded at 515 nm (FL 1) using a FACS Vantage® system (Becton-Dickinson, San Jose, CA, USA), and 10,000 events were counted per sample.

### Induction of Ovariectomy-Induced Osteoporosis

Female ICR mice (6 week-old) were used for this study. Mice received a sham operation (Sham, *n* = 10) or surgical ovariectomized (OVX, *n* = 20) under anesthesia. One week after surgery, the OVX mice were randomly divided into 2 groups of 10 mice each: bilateral OVX and bilateral OVX supplemented with 200 µl PBS containing 1 mM acteoside orally (AC group). Oral acteoside was administrated once every 3 days for 8 weeks after surgery, and the same amount of PBS was administered to the Sham and OVX groups. After 1 day of the last administration, mice were scarified and then biochemical parameters in serum and 3-dimensional bone structure were analyzed.

### Determination of Serum Biochemical Parameters

Blood samples were collected via cardiac puncture and serum was collected by centrifugation. Serum samples were stored at −80°C for analyses of biochemical parameters. The serum levels of IL-1β and IL-6 were estimated by using ELISA kit as described above, while alkaline phosphatase (ALP) activity was determined by using a biochemical colorimetric assay that measures the amount of *p*-nitrophenol produced from a *p*-nitrophenol phosphate substrate, as described elsewhere [27]. To estimate the biomarkers of bone formation and resorption, serum OC, calcium, and TRAP5b levels were also determined according to the manufacturers' instructions.

### Analyses of Bone Structure and Morphometric Parameters

The femora of mice (Sham, OVX, and AC groups) were dissected and filled with physiological saline for mechanical testing. The mechanical strength of the femur was measured as described elsewhere [28]. The fracture load was recorded as the peak force in newtons at the point that the mid shaft of the right femur fractured. In addition, the light femur of each animal was histomorphometrically analyzed using a microcomputer tomography (micro-CT) system (SkyScan 1076 microfocus X-ray system, Kontich, Belgium). In brief, the bones in 4% formaldehyde storage were dried superficially on paper tissue before being wrapped in plastic “cling-film” or in parafilm, to prevent drying during scanning. Each plastic-wrapped bones were placed in plastic/polystyrene foam tubes which were mounted vertically in the horizontally in the 1076 scanner sample chamber for micro-CT imaging. Scanning was carried out using 100 kV source voltage and 140 µA source current with 35 µm resolution. Three-dimensional models of the trabecular bones of femur were reconstructed using SkyScan CT Analyzer version 1.11. The structural parameters such as trabecular bone mineral density (BMD, g/cm^3^), percent bone volume (bone volume (BV)/tissue volume (TV), %), thickness (Tb.Th, µm), separation (Tb.Sp, mm), and number (Tb.N, 1/mm) were then measured.

### Osteogenic Differentiation and Mineralization Assay

Bone marrow cells cultured in 6-well culture plates were treated with DAG (10 nM dexamethasone, 50 µM ascorbic acid, and 20 mM β-glycerophosphate) in the presence of 10 µM acteoside. After 2 weeks of differentiation, the cells were fixed with ice-cold 70% (vol/vol) ethanol for 1 h and stained with 0.2% alizarin red S in distilled water for 30 min at room temperature. After the cells were destained and air-dried, the cell culture plates were evaluated by light microscopy using an inverted microscope (Nikon TS100, Japan). To quantify the amount of red dye, the stain was eluted with 10% acetylpyridinum chloride by shaking for 20 min and the absorbance was measured at 560 nm. The amount of calcium deposited in the cell layers was also measured using a Calcium C kit (Wako Chemical Inc. Osaka, Japan) according to the manufacturer's instructions. In addition, the expression of bone-specific mRNA markers, such as runt-related transcription factor-2 (Runx2), osterix, bone sialoprotein (BSP), and OC was determined by real-time RT-PCR. Oligonucleotide primers of these markers were designed with product sizes less than 200 bp using Primer Express Software 3.0 (Applied Biosystems) as described elsewhere [27].

### Statistical Analysis

Unless otherwise indicated, all data are expressed as the mean ± standard deviation (S.D.) of 3 or more independent experiments. A one-way ANOVA was used for multiple comparisons using SPSS version 12.0 software. A *p* value <0.05 was considered statistically significant.

## Results

### Acteoside Inhibits Osteoclast Formation by Macrophages in a Dose-Dependent Manner

**Figure 1:**
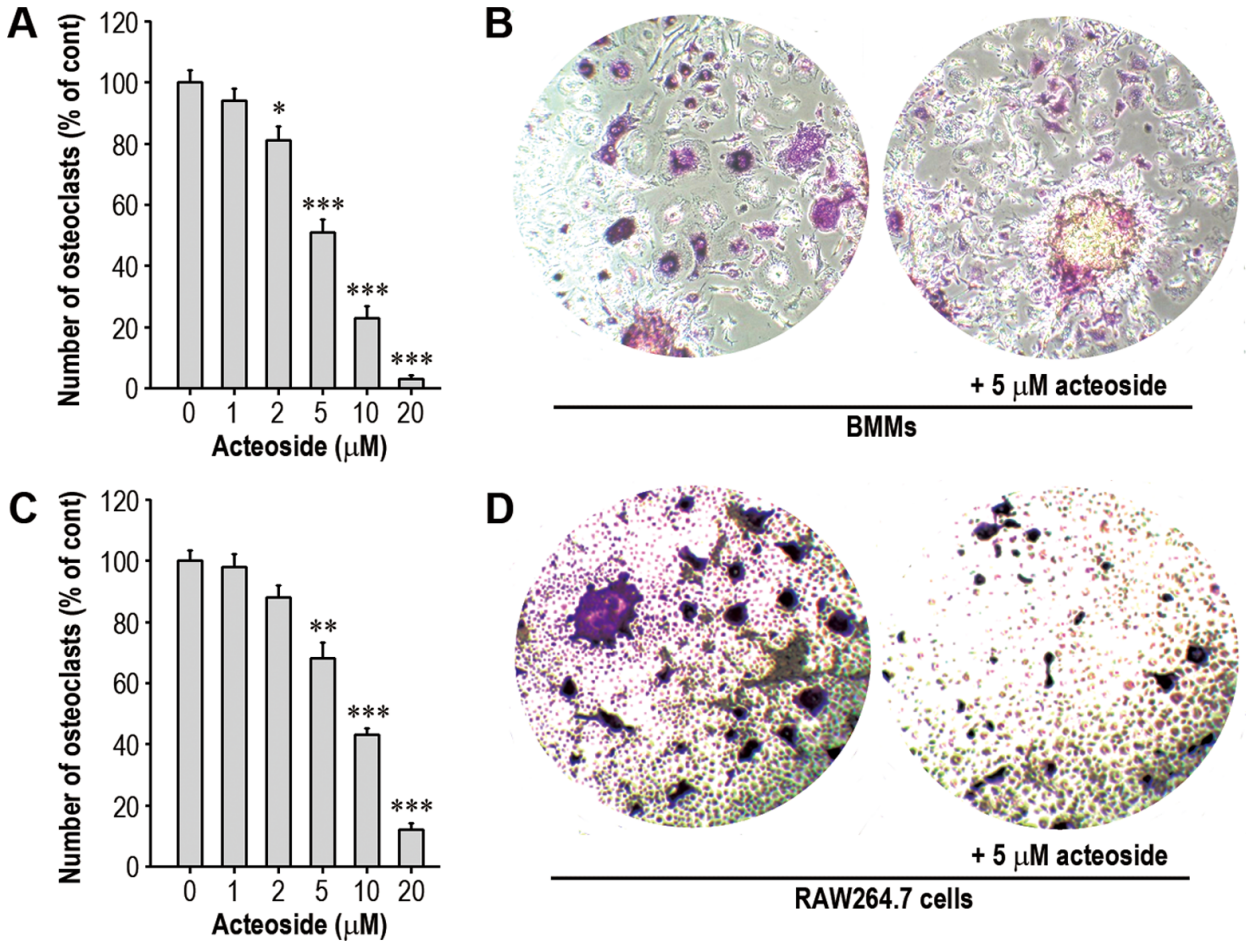
Acteoside inhibits RANKL-induced osteoclast differentiation from both BMMs and RAW264.7 cells. BMMs were cultured for seven days in the presence of M-CSF (50 ng/ml) and RANKL (100 ng/ml) with increasing concentrations (0–20 µM) (A) or 5 µM acteoside (B). C and D, RAW264.7 cells were also exposed to the indicated acteoside concentrations in the presence of 100 ng/ml RANKL for seven days. After culturing, these cells were TRAP stained and the number of osteoclasts was counted. In the panels A and C, the results are expressed as a percentage of osteoclasts generated by M-CSF+RANKL (for BMMs) or RANKL alone (for RAW264.7 cells). Data are representative of three independent experiments (*n* = 4 per experiment). ^*^ *p*<0.05, ^**^ *p*<0.01, and ^***^ *p*<0.001 vs. the cells cultured without acteoside.

**Figure 2:**
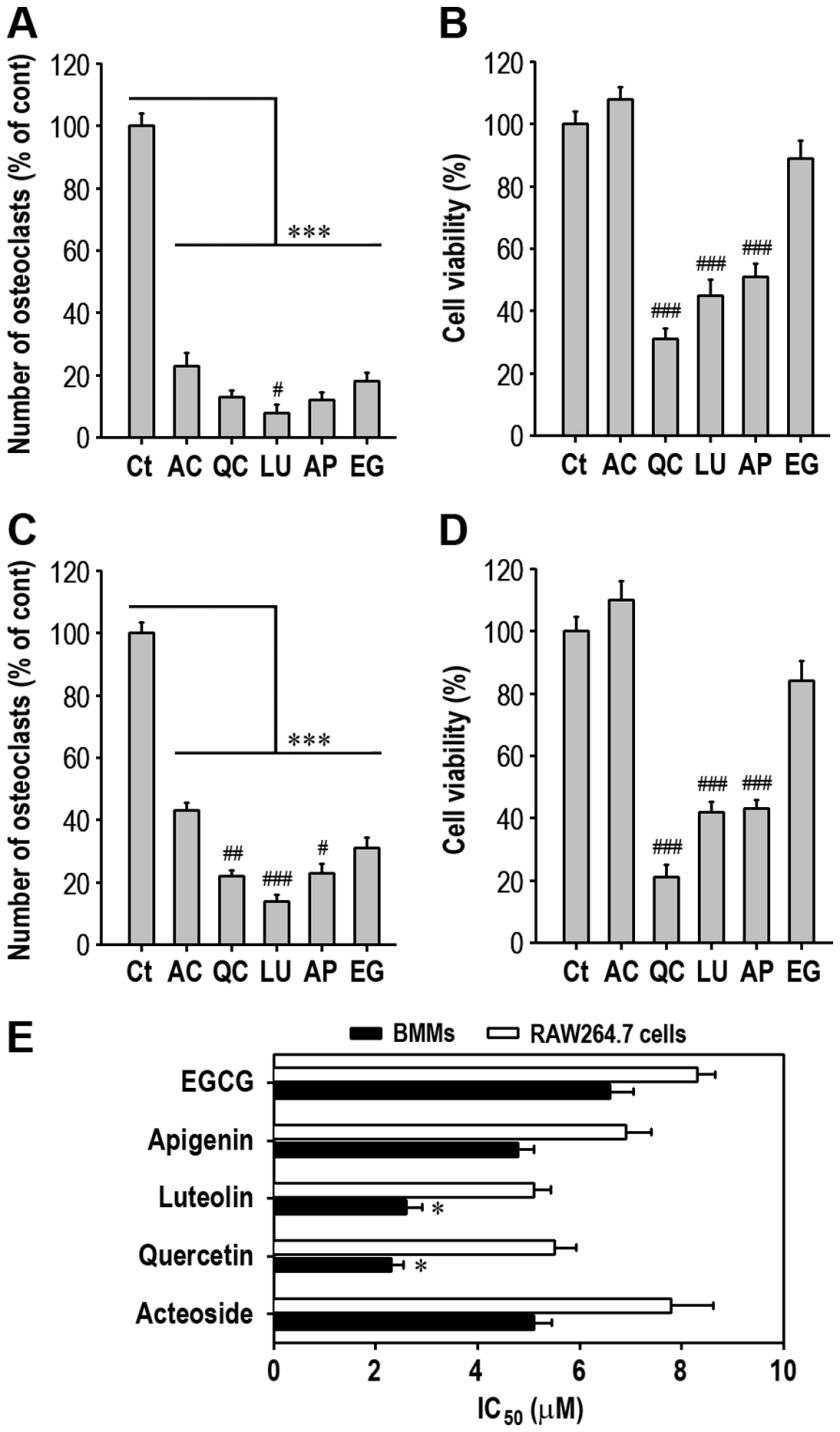
Acteoside attenuates RANKL-induced osteoclast differentiation without cytotoxic effects. BMMs (A) or RAW264.7 cells (C) were cultured for 7 days in the presence of 50 ng/ml M-CSF, 100 ng/ml RANKL, or both with 10 µM of an anti-resorptive compound. TRAP staining was performed and the number of osteoclasts generated was calculated from 3 independent experiments (3 wells per condition were counted in each experiment). In addition, BMMs (B) and RAW264.7 cells (D) were incubated with 10 µM of each compound for 48 h and cell viability was measured by using WST-8 regent (*n* = 5 per each experiment). E, The concentration of the compounds to inhibit 50% of osteoclast formation was calculated from triplicate experiments. ^*^ *p*<0.05 vs. acteoside. ^***^ *p*<0.001 vs. controls cultured with M-CSF and/or RANKL only. ^#^ *p*<0.05, ^##^ *p*<0.01, and ^###^ *p*<0.001 vs. cells cultured with acteoside. AC, acteoside; QC, quercetin; LU, luteolin; AP, apigenin; EG, EGCG.

To verify the effect of acteoside on BMM differentiation into osteoclasts, the cells were cultured with various concentrations (0–20 µM) of acteoside for 7 days in the presence of 50 ng/ml M-CSF and 100 ng/ml RANKL. Acteoside reduced the number of osteoclasts in a dose-dependent manner. When the cells were pretreated with 10 µM acteoside for 2 h, the osteoclast number decreased by 43% compared to cells supplemented with M-CSF and RANKL (Fig. 1A). Fig. 1B shows the RANKL-mediated osteoclast differentiation and its inhibition by combined treatment with acteoside. Consistent with this result, acteoside pretreatment decreased the RANKL-stimulated differentiation of RAW264.7 cells and osteoclast formation (Figs. 1C and D). When the anti-osteoclastic potential of acteoside on BMMs was compared with the anti-osteoclast potentials of several phenolic compounds at the same concentration (10 µM), luteolin showed the highest activity (Fig. 2A). However, luteolin, quercetin, or apigenin itself decreased viability of the cells (Fig. 2B). All the compounds inhibited osteoclastic differentiation by RAW264.7 macrophages with the following relative activities: luteolin > quercetin  =  apigenin > EGCG  =  acteoside (Fig. 2C). Luteolin, quercetin, or apigenin itself also showed the decreased viability of the cells (Fig. 2D). In contrast, quercetin treatment only caused a significant reduction of viability in both BMMs and RAW264.7 cells, when these cells were exposed to 10 µM of each compound for 2 days with 50 ng/ml M-CSF, 100 ng/ml RANKL or both (data not shown). When the concentration of these compounds required to inhibit 50% of osteoclast formation in BMMs (IC_50_) was calculated using concentration-activity curves, the IC_50_ of acteoside, quercetin, luteolin, apigenin, and EGCG was 5.1, 2.3, 2.6, 4.8 and 6.6 µM, respectively (Fig. 2E). This result was similar to the case that RAW264.7 macrophages were examined. These data suggested that luteolin and quercetin had anti-clastogenic activities higher than acteoside. In contrast, quercetin at the IC_50_ also had a mild toxic effect on the cells (data not shown).

### Acteoside Inhibits Bone Resorption by Macrophages

**Figure 3:**
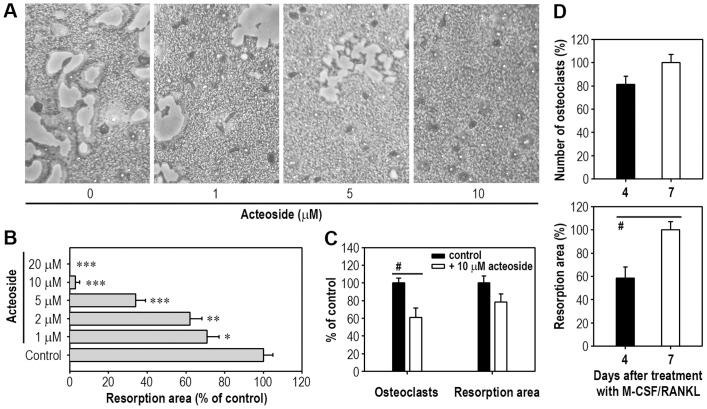
Acteoside prevents RANKL-induced pit formation in BMMs. A, BMMs were pretreated with the indicated doses of acteoside for 2-coated 24-well plates and stimulated with 50 ng/ml M-CSF and 100 ng/ml RANKL for 7 days. Pit formation on the plate was observed under optic microscopy. B, BMMs were also cultured with M-CSF and RANKL in the presence of various acteoside concentrations (0–20 µM), and 7 days later, the resorbed area was quantified from 3 independent experiments and expressed as a percentage of the control (*n* = 4 per experiment). C, BMMs were treated with 10 µM acteoside 4 days after M-CSF and RANKL stimulation and incubated for addition-3 days followed by the analyses for TRAP staining and pit formation. The results in panel D show osteoclast and pit formation in BMMs 4 and 7 days after the osteoclastogenic induction without supplementation of acteoside. ^*^ *p*<0.05, ^**^ *p*<0.01, and ^***^ *p*<0.001 vs. cells cultured with M-CSF and RANKL. ^#^ *p*<0.05 indicates significant difference between the experiments.

Acteoside also prevented RANKL-mediated bone resorption in a dose-dependent manner, as measured by an *in vitro* model system (Fig. 3A). Bone resorption was significantly inhibited when BMMs were incubated with 1 µM acteoside (Fig. 3B). A 10 µM acteoside treatment almost completely attenuated RANKL-induced pit formation by BMMs. Similarly, acteoside decreased bone resorption in RANKL-stimulated RAW264.7 cells (Figs. S2A and B). The ability of acteoside to inhibit bone resorption depended on the timing of the treatment relative to RANKL stimulation. Acteoside (10 µM) added 4 days after RANKL stimulation did not reduce pit formation in BMMs, whereas it suppressed the number of osteoclasts formed (Fig. 3C). This different result was in part due to the pit area already formed after 4 days of RANKL stimulation formation (Fig. 3D).

### Acteoside Down-Regulates Early RANKL Signaling Pathways

**Figure 4:**
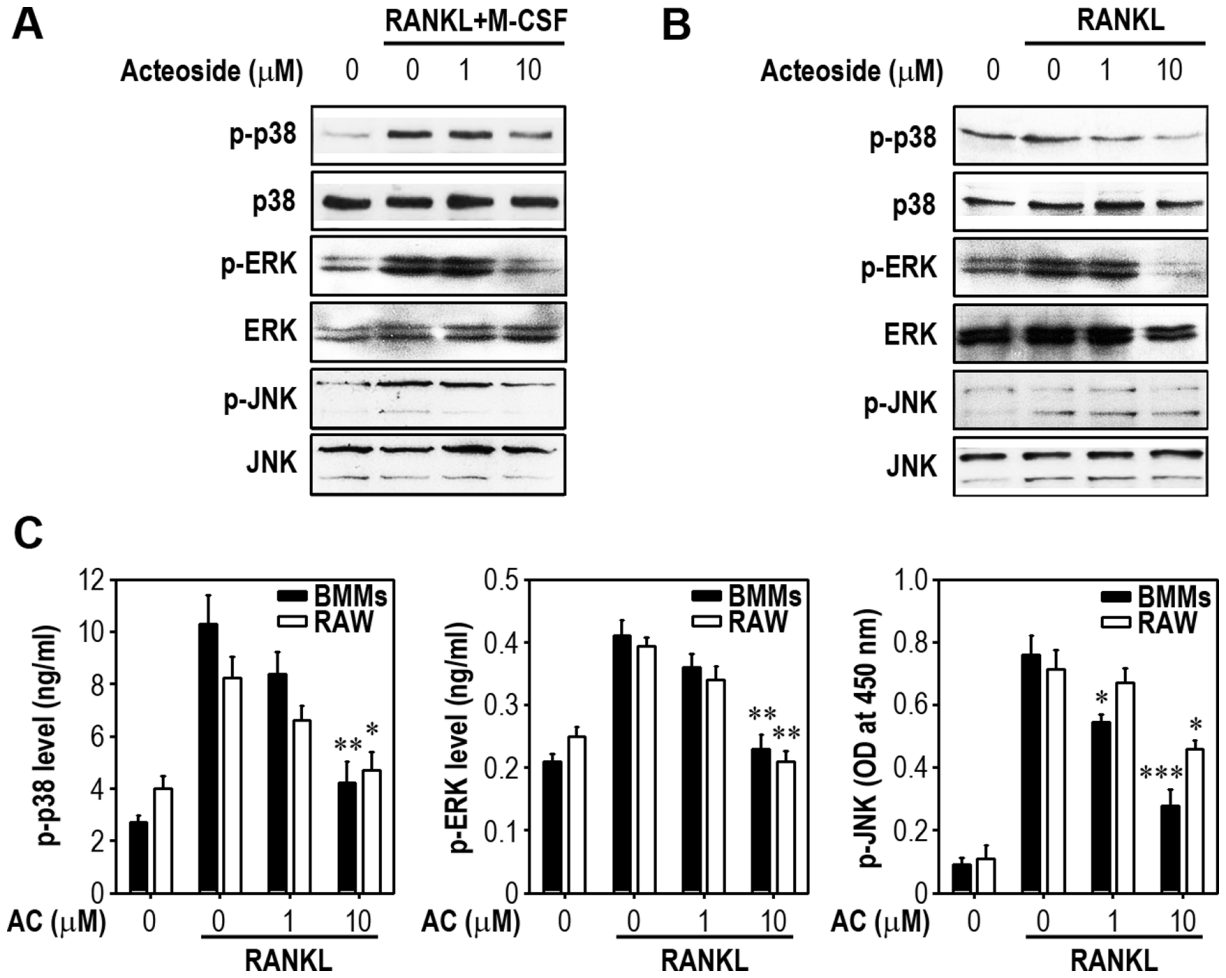
Acteoside inhibits RANKL-induced MAPK activation in both BMMs and RAW264.7 cells. BMMs (A) and RAW264.7 cells (B) were pretreated with the increasing doses (0–10 µM) of acteoside for 2 h followed by stimulation with 50 ng/ml M-CSF, 100 ng/ml RANKL, or both for 30 min. The phosphorylation of p38, ERK, and JNK was determined by immunoblot analysis using specific antibodies. The representative results from triplicate experiments are shown. C, Cells were stimulated with RANKL in the presence of acteoside and an immunometric assay was used to determine MAPK activities. The results were calculated from 3 independent experiments and are expressed as ng/ml (for p-p38 and p-ERK) or optical density (OD) at 450 nm (for p-JNK) normalized to control values (*n* = 4 per experiment). ^*^ *p*<0.05, ^**^ *p*<0.01, and ^***^ *p*<0.001 vs. cells stimulated with RANKL.

**Figure 5:**
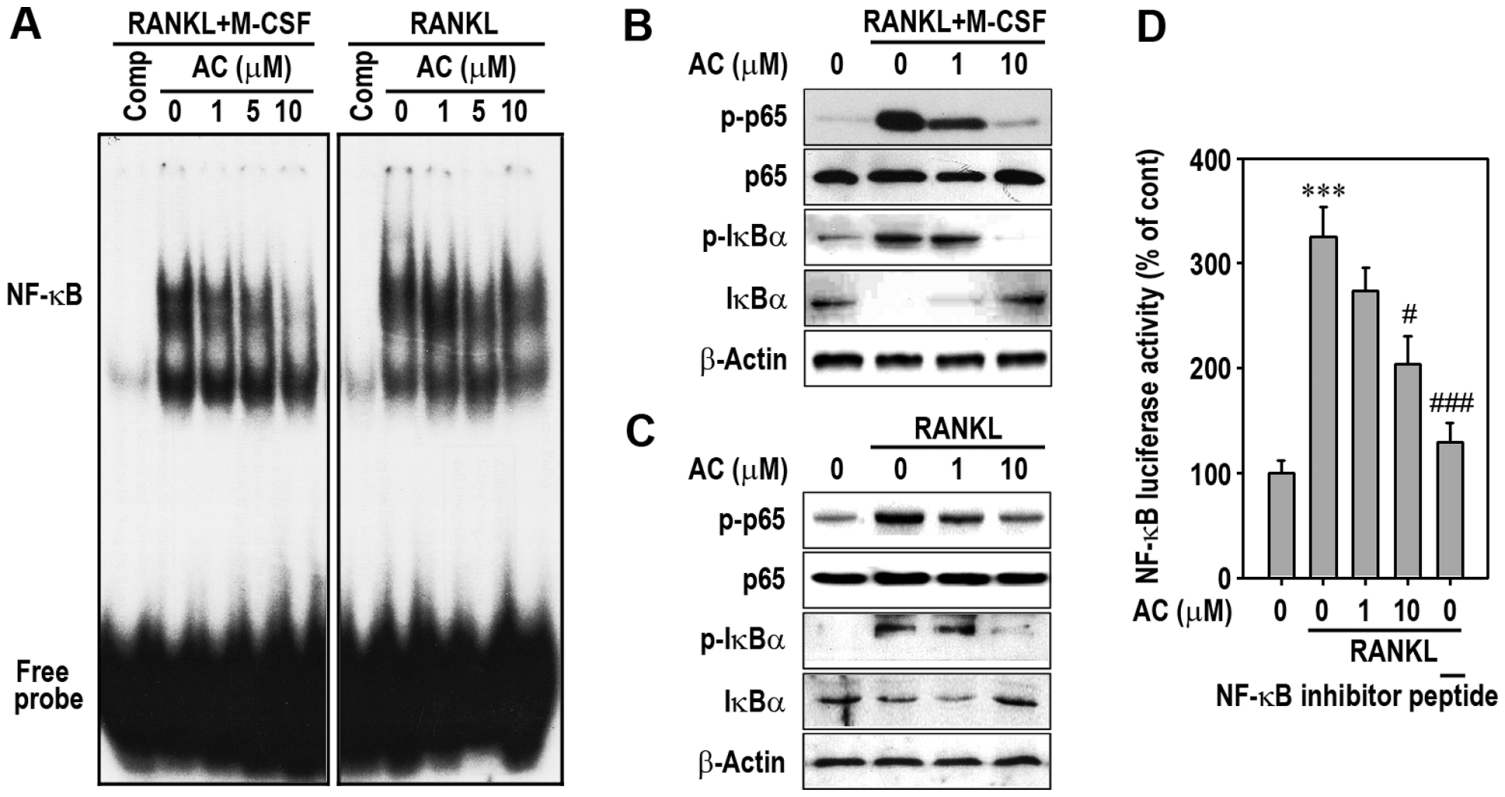
Acteoside suppresses NF-κB-DNA binding and phosphorylation of IκBα and the p65 subunit in RANKL-stimulated macrophages. BMMs and RAW264.7 cells were pretreated with the indicated doses of acteoside for 2/ml RANKL for 30 min. A, NF-κB-DNA binding activity was determined and a representative result from triplicate experiments is shown. The phosphorylation of p65 and IκBα from BMMs (B) and RAW264.7 cells (C) was analyzed by immunoblotting. D, RAW264.7 cells were stimulated with 100 ng/ml RANKL for 24 h in the presence of 1 or 10 µM acteoside, and luciferase activity was measured. The result was calculated from 3 independent experiments and is expressed as a percentage of the control activity (*n* = 4 per experiment). ^***^ *p*<0.001 vs. cells without RANKL or acteoside. ^#^ *p*<0.05 and ^###^ *p*<0.001 vs. cells stimulated with RANKL alone. An NF-κB inhibitor peptide was used as a positive control.

RANKL induces the activation of 3 well-known MAPKs and NF-κB in osteoclast precursors, and this activation is required for early osteoclast differentiation. To understand the possible mechanisms by which acteoside inhibits osteoclastogenesis, we investigated the effect of acteoside on MAPKs and NF-κB activation in macrophages. BMMs and RAW264.7 cells were pretreated with 10 µM acteoside for 2 h and then stimulated with 100 ng/ml RANKL for 30 min. MAPK phosphorylation was examined by Western blotting and immunometric analysis. RANKL induced phosphorylation of p38, ERK, and JNK in BMMs (Fig. 4A) and RAW264.7 cells (Fig. 4B). Acteoside prevented these RANKL-induced increases in p-p38, p-ERK, and p-JNK. This result was supported by immunometric analysis, which that pretreatment with 10 µM acteoside significantly inhibited the levels of phosphorylated MAPKs in these macrophages (Fig. 4C). RANKL treatment increased the DNA-binding of NF-κB, whereas acteoside inhibited RANKL-induced activation of NF-κB-DNA binding (Fig. 5A). This inhibition was more prominent in BMMs than in RAW264.7 cells. Acteoside also diminished RANKL-stimulated p65 and IκBα phosphorylation in BMMs and RAW264.7 cells (Figs. 5B and C). Adding 10 µM acteoside almost completely inhibited both the degradation and activation of IκBα in BMMs (Fig. 5B). To further confirm that NF-κB activation is involved in the action of acteoside, κB promoter-luciferase constructs were transiently transfected into RAW264.7 cells. The cells incubated with 100 ng/ml RANKL had 3-fold higher κB promoter activity, which was significantly attenuated by 10 µM acteoside (Fig. 5D).

### Acteoside Suppresses the Production of Inflammatory Cytokines and the Expression of TNF-α, c-Fos and NFATc1 in RANKL-Stimulated Macrophages

**Figure 6:**
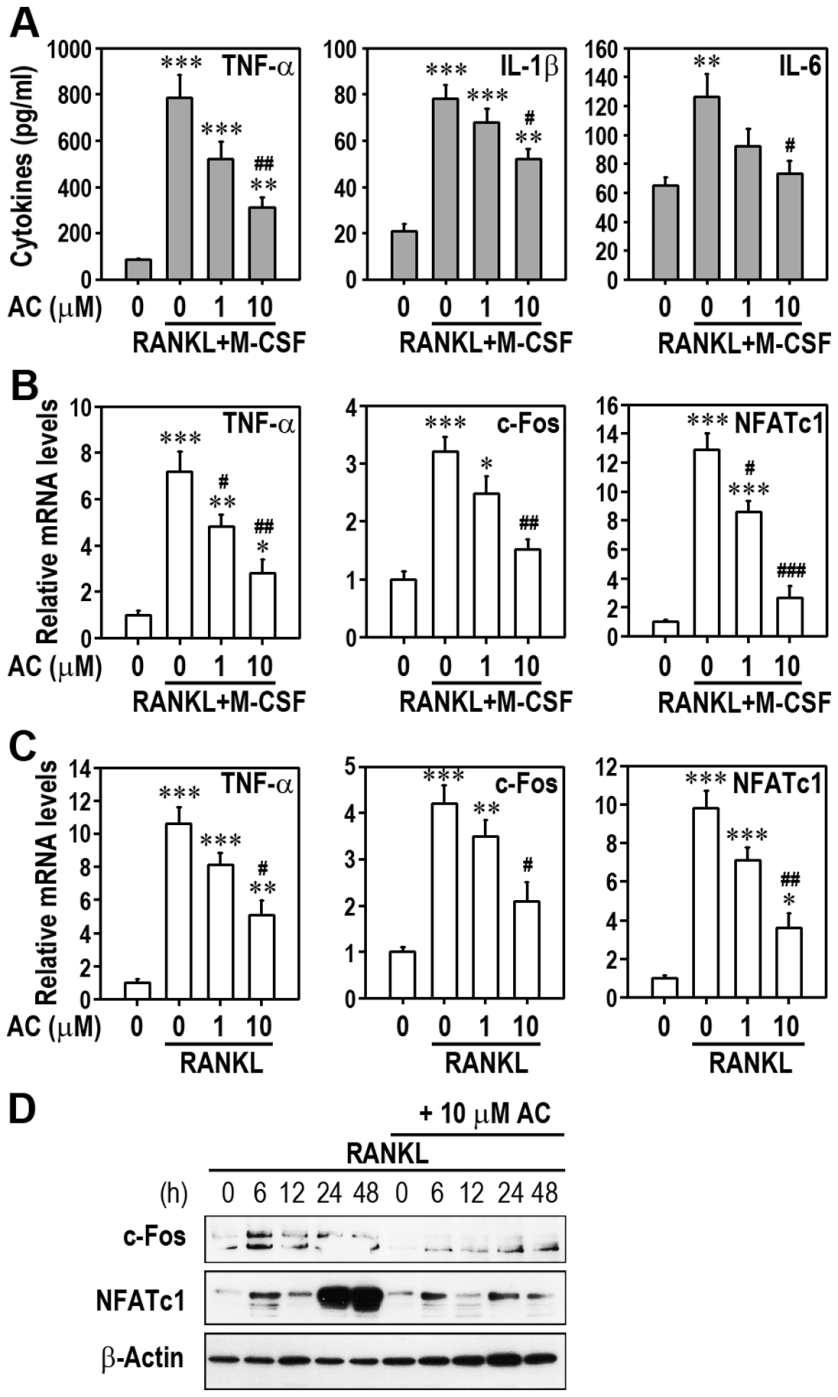
Acteoside attenuates inflammatory cytokine production and expression of c-Fos and NFATc1 in RANKL-stimulated macrophages. A, BMMs were pretreated with increasing concentrations (0–10 µM) of acteoside for 2 h followed by stimulation with 50 ng/ml M-CSF and 100 ng/ml RANKL for 48 h. The levels of TNF-α, IL-1β, and IL-6 were determined using ELISA kits. BMMs (B) and RAW264.7 cells were also stimulated with 100 ng/ml RANKL in the presence of acteoside for 24 h and subjected to real-time RT-PCR analysis. In panels A, B, and C, the results were calculated from 3 independent experiments and are expressed as pg/ml or mRNA levels relative to the control (*n* = 4 per experiment). ^*^ *p*<0.05, ^**^ *p*<0.01, and ^***^ *p*<0.001 vs. cells without RANKL and acteoside. ^#^ *p*<0.05, ^##^ *p*<0.01, and ^###^ *p*<0.001 vs. cells stimulated with RANKL. D, BMMs were pretreated with 10 µM acteoside for 2 h and stimulated with 100 ng/ml RANKL. At the indicated times (0–48 h after RANKL stimulation), c-Fos and NFATc1 protein levels were determined by Western blotting.

TNF-α, IL-1β, and IL-6 are important in osteoclast formation and function, which is mediated by NF-κB signaling in RANKL-stimulated macrophages. RANKL stimulated the production of these cytokines, and this production was markedly reduced by 10 µM acteoside pretreatment in BMMs (Fig. 6A). Similarly, acteoside attenuated the RANKL-induced production of cytokines, except IL-6, in RAW264.7 macrophages (Fig. S3). To understand the molecular mechanisms of acteoside action in osteoclastogenesis, we further examined the effect of acteoside on TNF-α, c-Fos, and NFATc1 expression. RANKL up-regulated the mRNA expression of these factors in BMMs and RAW264.7 cells (Figs. 6B and C). Pretreatment with 10 µM acteoside significantly inhibited the RANKL-induced expression of these factors in both BMMs and RAW264.7 cells. Acteoside pretreatment also strongly reduced the protein levels of c-Fos and NFATc1 in RANKL-stimulated BMMs (Fig. 6D). These results suggest that acteoside down-regulates RANKL inducing mediators of osteoclast formation at the gene and protein levels.

### Acteoside Diminishes Intracellular ROS Generation in BMMs in a Dose-Dependent Manner

**Figure 7:**
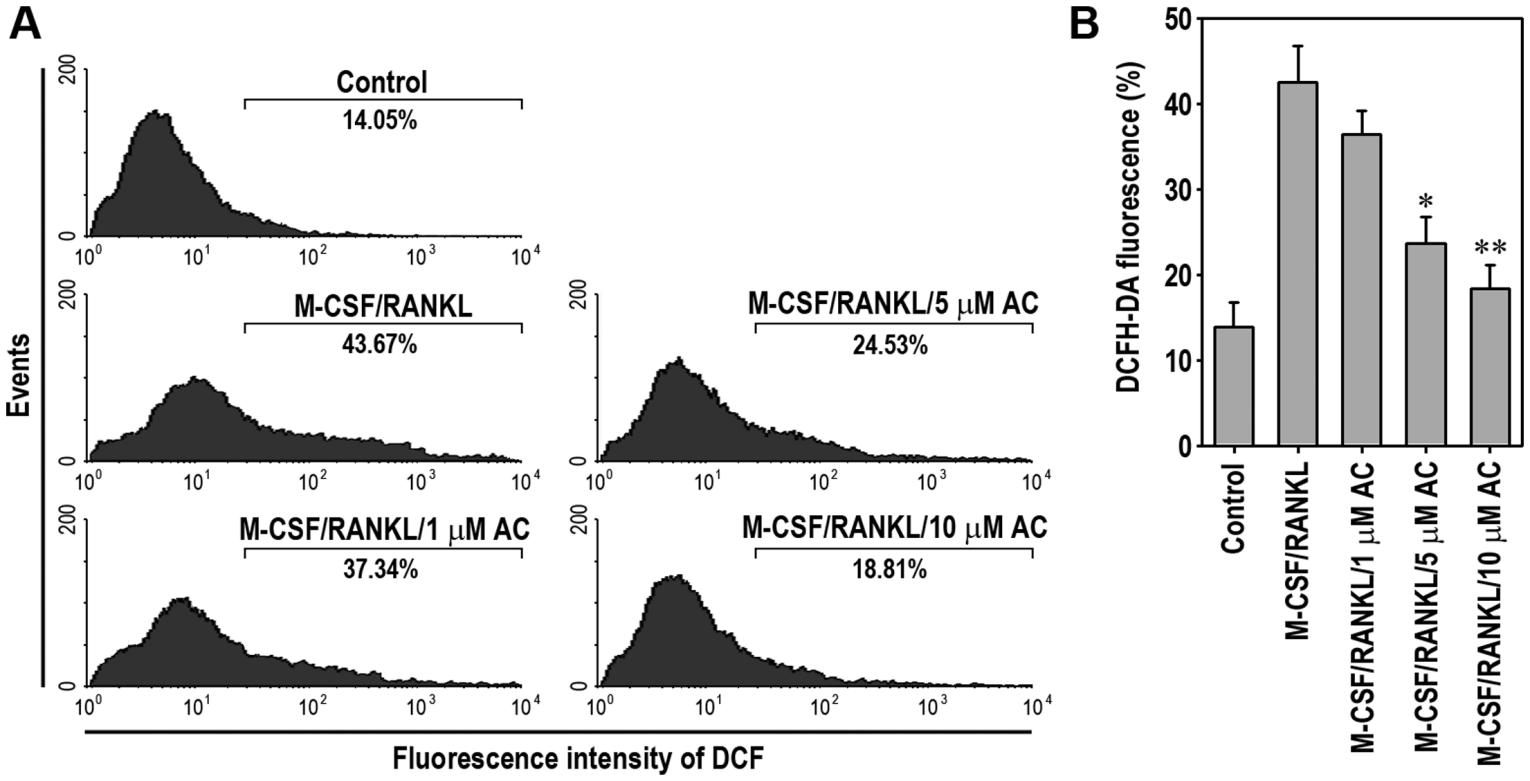
Acteoside inhibits RANKL-mediated ROS production on osteoclast differentiation in BMMs. The cells were incubated with 50/ml M-CSF for 24 h and then stimulated with 100 ng/ml RANKL for 1 h in the presence and absence of the indicated concentrations of acteoside. A, Cellular ROS were determined using flow cytometric analysis. B, DCF intensity was also calculated using WinMDI 2.9 program (*n* = 3). ^*^ *p*<0.05 and ^**^ *p*<0.01 vs. cells stimulated with M-CSF/RANKL.

Since it is known that intracellular ROS production is correlated with RANKL-stimulated osteoclastogenesis, we investigated whether acteoside inhibits ROS production during RANKL-mediated osteoclast differentiation using a cell-permeable, oxidation-sensitive dye, DCFH-DA. Flow cytometry analysis showed that the mean fluorescence signal specific to DCF in BMMs was apparently right-shifted after stimulation with RANKL, compared to the un-treated control cells (Fig. 7A). This shift was similar to the case that RAW264.7 macrophages were observed after RANKL stimulation (data not shown). Treating BMMs with acteoside reduced the signal intensity of the DCF in a dose-dependent manner. Pretreatment with 10 µM acteoside almost completely reduced the levels of intracellular ROS produced during osteoclast differentiation to the un-treated control levels (Fig. 7B).

### Oral Acteoside Administration Inhibits Alteration of Osteoporotic Biochemical Markers and Bone Loss in Ovariectomized Mice

**Figure 8:**
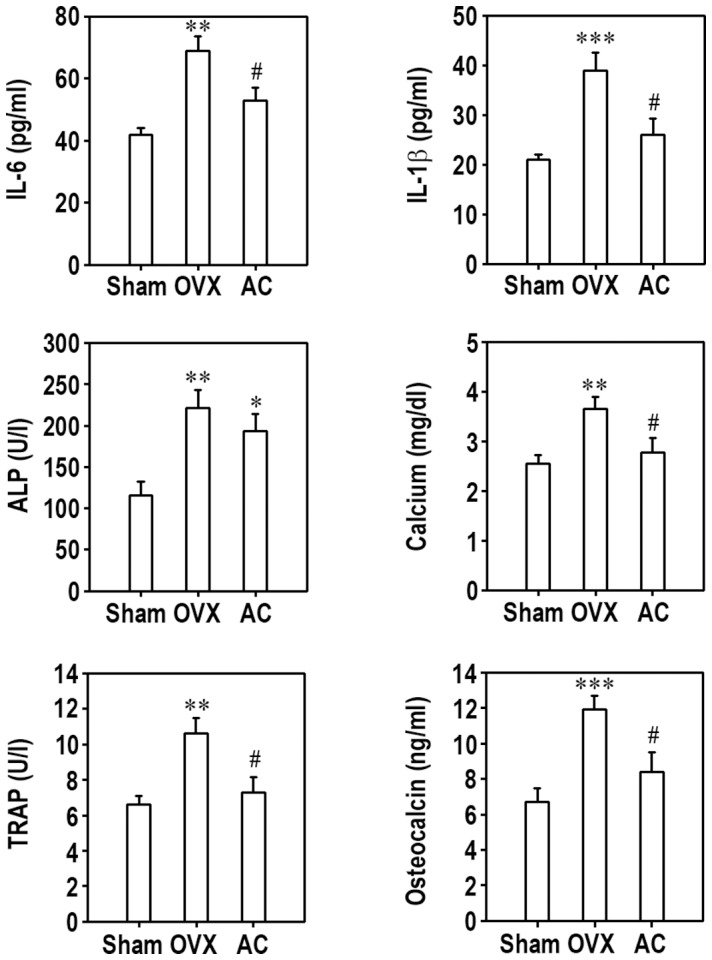
Oral administration attenuates the increases in serum biomarkers of bone turnover in ovariectomized animals. Female ICR mice were ovariectomized (OVX) or given a sham operation (Sham). OVX mice were orally supplemented with 200 µl PBS containing 1 mM acteoside (AC mice) for 8 weeks and Sham and OVX mice were given the same volume of PBS for the same period. The serum levels of IL-6, IL-1β, ALP, calcium, TRAP, and OC were determined. Data are presented as mean ± SD (*n* = 8–10 mice/group). ^*^ *p*<0.05, ^**^ *p*<0.01, and ^***^ *p*<0.001 vs. Sham group. ^#^ *p*<0.05 vs. OVX group.

**Figure 9:**
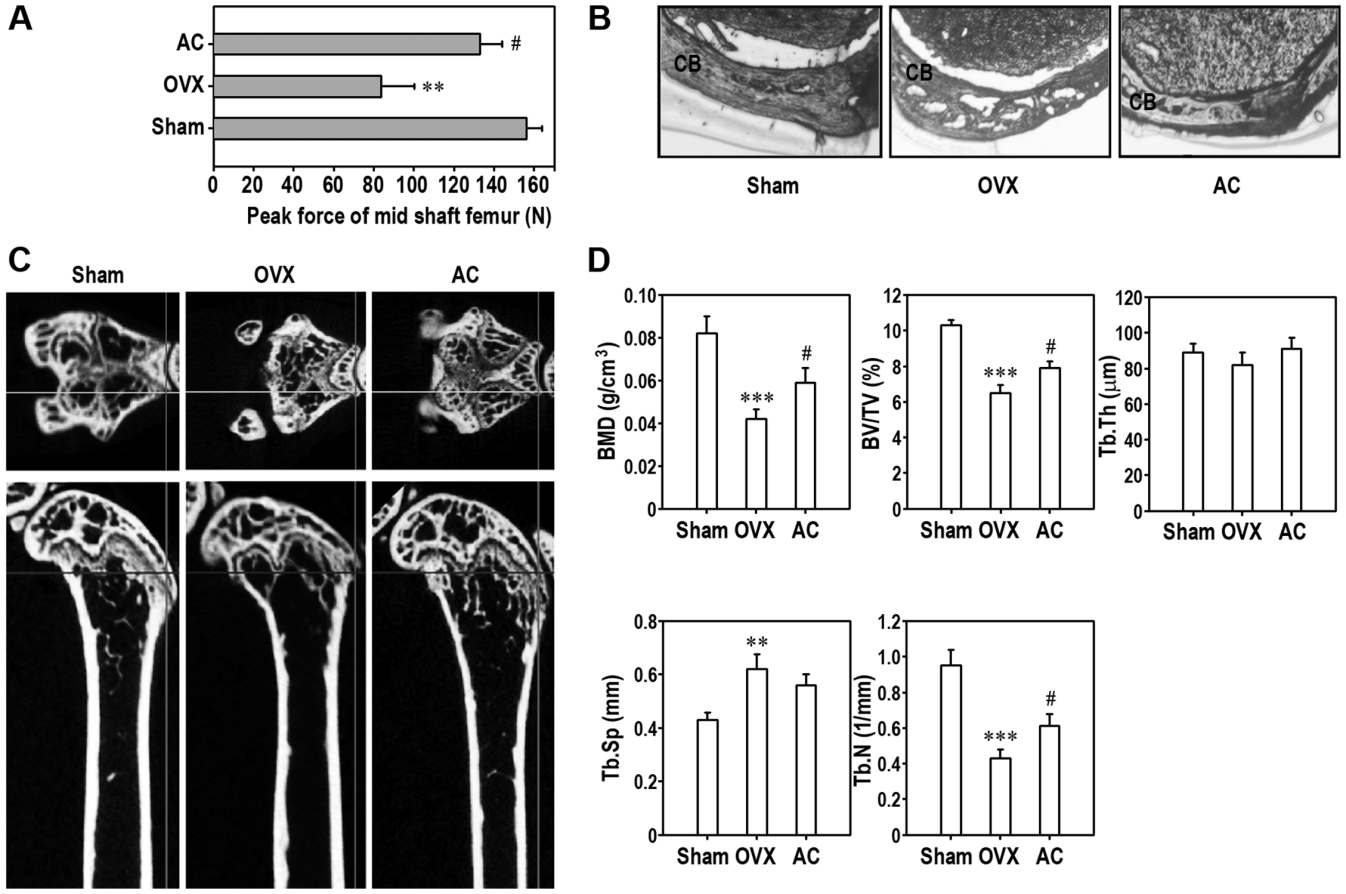
Acteoside restores fracture maximum force of the right mid-shaft of the femur and inhibits trabecular bone loss in ovariectomized animals. At 1 day after the last acteoside treatment, the light femur of animals was collected and processed for various physiologic and morphometric analyses. A, Peak fracture force of mid-shaft of the femur was determined and expressed as ‘N’. B, Cortical bone of right femur was observed by optic microscopy. CB, cortical bone. C, Micro-CT images of the proximal femur. D, BMD (g/cm^3^), BV/TV (%), Tb.Th (µm), Tb.Sp (mm), and Tb.N (1/mm) were analyzed with micro-CT SkyScan CTAn software. Data are presented as mean ± SD (*n* = 8–10 mice/group). ^**^ *p*<0.01 and ^***^ *p*<0.001 vs. Sham group. ^#^ *p*<0.05 vs. OVX group.

To explore the effect of acteoside on bone loss, we prepared an osteoporotic animal model by ovariectomy. There was no significant difference of body weight between OVX and Sham mice during the experimental period (data not shown). The OVX group had significant higher serum levels of IL-1β and IL-6 than the Sham group (Fig. 8). Ovariectomy-induced increases in these inflammatory cytokines were attenuated by oral acteoside administration (AC group). The serum levels of bone turnover markers such as ALP, calcium, TRAP5b, and OC were significantly increased in OVX group. Of these osteoporotic markers, the increased levels of calcium, TRAP5b, and OC in OVX mice were apparently inhibited by acteoside treatment, whereas serum level of ALP was not changed by the treatment. The average maximum fracture load to the middle of the right femoral shaft was significantly lower in the OVX group than in the Sham group (Fig. 9A). Acteoside treatment raised the maximum fracture back up to that of the Sham group. When the cortical bone of the femur was dissected and observed by optic microscopy, the osteoporotic features shown in OVX group had almost completely disappeared in AC mice (Fig. 9B). To verify the effect of acteoside on OVX-induced osteoporosis model, BMD and bone morphologic parameters in trabecular of the light proximal femur were analyzed by micro-CT. As shown in Fig. 9C, an alteration of the femoral trabecular architecture was found in OVX mice, whereas this change was diminished by treatment with acteoside. The results from micro-CT analysis revealed that BMD, a measure of bone strength, was dramatically reduced in OVX mice (Fig. 9D). Compared to Sham group, OVX mice also showed significant changes in BV/TV, Tb.Sp, and Tb.N, but not in Tb.Th. Oral acteoside treatment of OVX mice significantly prevented the alteration in BMD as well as BV/TV and Tb.N.

### Acteoside does not Affect Osteoblastogenesis in Bone Marrow Cells

**Figure 10:**
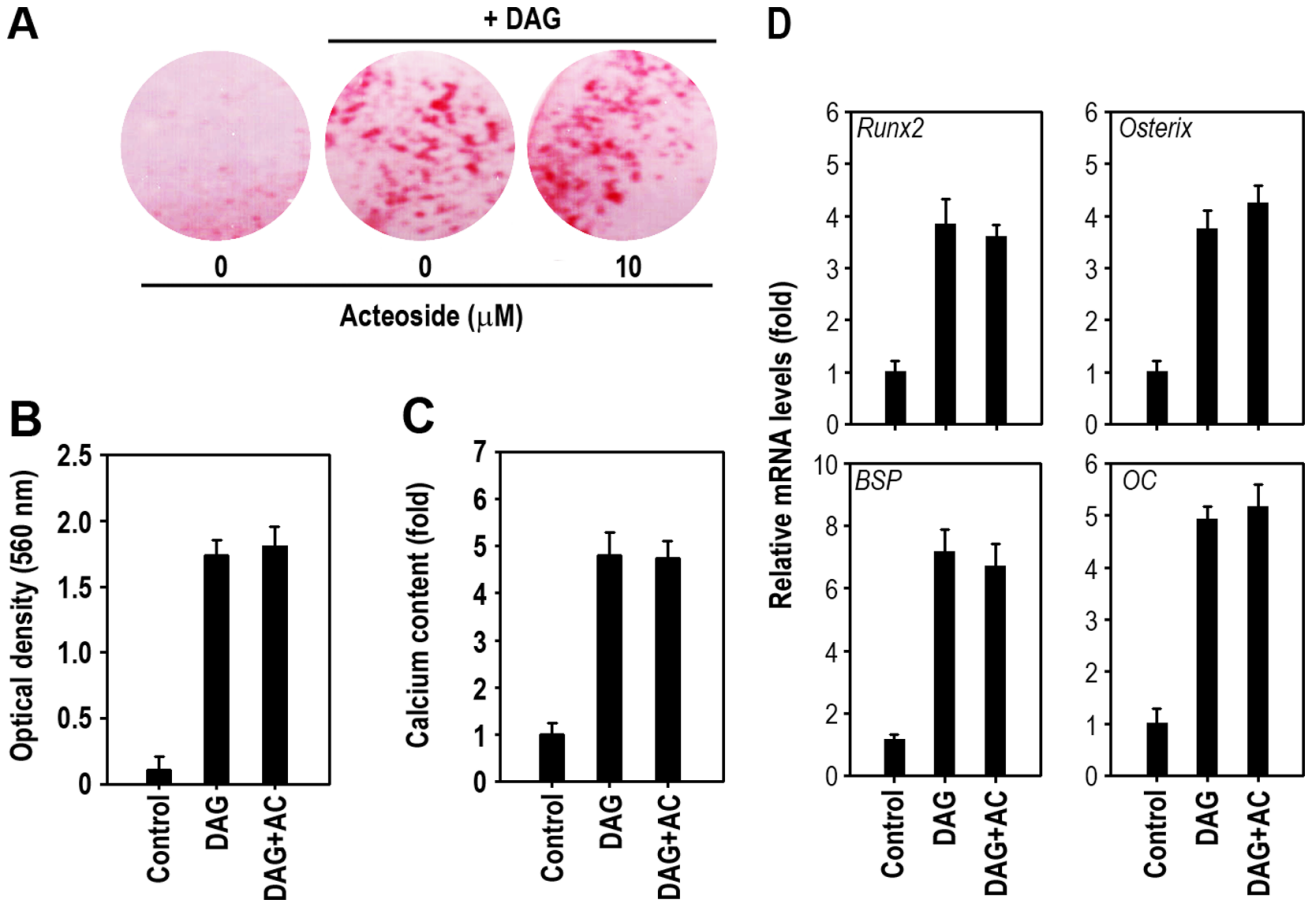
Acteoside does not affect osteoblastogenesis of bone marrow cells. A, Bone marrow cells were pretreated with 10 µM acteoside followed by treatment with DAG for 2 weeks and then stained with alizarin red. The dye absorbance (B) and calcium accumulation (C) were determined at the same time. D, After 2 days of DAG treatment, the mRNA levels of *Runx2*, *osterix*, *BSP*, and *OC* were measured by real-time RT-PCR. The results in panels B, C, and D was calculated from 3 independent experiments (*n* = 4 per experiment) and expressed as optical density or fold increase as compared to the controls without DAG or acteoside.

The role of acteoside on osteoblastic differentiation was further investigated using bone marrow cells. As shown in Fig. 10A, DAG treatment increased the number of alizarin red-stained cells and this was not changed by 10 µM acteoside pretreatment. The amount of dye present indicated that the combined acteoside and DAG treatment did not change mineralization (Fig. 10B). Similarly, DAG-induced increases in the intracellular calcium content (Fig. 10C) and mRNA levels (Fig. 10D) of bone specific markers, such as Runx2, osterix, BSP, and OC, were not affected by pretreatment with acteoside.

## Discussion

Bone remodeling is tightly regulated by the balance between bone formation by osteoblasts and bone resorption by osteoclasts. Prolonged and excessive bone resorption causes imbalance of skeletal turnover, resulting in bone-resorptive diseases. To explore the effects of acteoside on osteoclastogenesis, we used two macrophages, primary cultured BMMs and RAW264.7 cells. These cells were stimulated with RANKL to differentiate into osteoclasts in the presence and absence of acteoside. We showed for the first time that acteoside inhibits osteoclast differentiation and formation. Acteoside itself at the concentrations examined did not cause a decrease of viability of the primary cultured macrophages in both conditions of growth and differentiation. Acteoside treatment also decreased the resorption activity of mature osteoclasts. These results suggest that acteoside suppresses osteoclastic formation from macrophages and osteoclast resorption activity. The results from our culture system, which did not include osteoblasts or stromal cells, also suggest that acteoside prevents osteoclast formation by directly acting on osteoclast precursors.

RANKL activates MAPKs including p38, ERK, and JNK. These three kinases are involved in early osteclastic differentiation and thus their inhibition pharmacologically or with a dominant-negative JNK transfection suppresses RANKL-induced osteoclastogenesis [29]. Our results revealed that acteoside pretreatment inhibited all these kinases, indicating a non-specific down-regulation of MAPKs. This result differed in part from previous report that EGCG, the major anti-inflammatory compound in green tee, specifically attenuated JNK activation without affecting ERK or p38 activation in RANKL-stimulated BMMs [7]. Paeonol, an anti-inflammatory compound derived from a Chinese herb, has also been reported to inhibit ERK and p38, but not JNK, phosphorylation in RANKL-stimulated RAW264.7 cells [30]. In contrast, silibinin, a novel inhibitor in bone, attenuated RANKL-induced activation of p38, ERK, and JNK [31]. These findings suggest that the effects of anti-resorptive compounds on MAPK activation by RANKL depend on the compound, though all three MAPKs are involved in early osteoclastogenesis. Considering the observation that acteoside attenuated p-JNK levels in RANKL-stimulated BMMs, even at 1 µM, the blockage of JNK rather than of p38 MAPK or ERK appeared to be more specific event in acteoside-mediated anti-osteoclastogenesis in the cells. Although acteoside at the same concentration did not reduce the number of osteoclasts in BMMs, there was a significant decrease in pit formation by acteoside treatment. We also found that pretreatment with SP600125, a pharmacological inhibitor specific to JNK, dramatically prevented the formation of osteoclasts (data not shown). Collectively, these findings suggest that JNK-mediated signaling is closely related to the acteoside-mediated suppression of osteoclastogenesis stimulated by RANKL.

NF-κB signaling regulates cellular events, including apoptosis, cell-cycle progression, cell adhesion, cytokine production, and survival in macrophages [32]. NF-κB signaling is also required for osteoclast development, which has been demonstrated by the appearance of osteopetrosis in NF-κB-knockout mice [33], [34]. Therefore, inhibiting NF-κB is proposed to be an effective target for anti-resorptive agents to down-regulate osteoclast activity and treat osteoporosis. Post-translational modification of NF-κB subfamily proteins is crucial in modulating NF-κB activity. Especially, phosphorylation of the p65 subunit and IκB kinase is critical for NF-κB to induce osteoclastogenesis [7]. Our present findings showed that RANKL stimulation increased DNA binding activity of NF-κB and phosphorylation of the p65 subunit and IκBα in both BMMs and RAW264.7 cells. Pretreatment with acteoside inhibited these RANKL-induced increases, resulting in down-regulated NF-κB activity. Consequently, these results suggest that, in addition to MAPKs, NF-κB signaling is the main target of acteoside in inhibiting osteoclast differentiation and formation from RANKL-stimulated macrophages.

In addition to NF-κB signaling, the c-Fos/c-Jun/NFATc1 pathway plays key roles in osteoclast development, thus the lack of any of these proteins can arrest osteoclastogenesis [35], [36]. In this study, we found that acteoside prevented the RANKL-induced c-Fos and NFATc1 expression at the mRNA and protein levels. JNK is an upstream kinase of c-Jun, which is required for NFATc1 expression and osteoclastogenesis in response to RANKL [29]. Blocking the JNK/c-Jun pathway with a JNK inhibitor diminished RANKL-induced osteoclast formation and c-Fos and NFATc1 expression [7]. Our results and previous findings suggest that inhibiting JNK-mediated signaling by acteoside is closely associated with preventing RANKL-mediated c-Fos and NFATc1 expression, which suppresses osteoclast differentiation in macrophages. Differences in the effects of acteoside on BMMs and RAW264.7 cells are at least in part due to differences in the sensitivity to JNK inhibition.

TNF-α can induce osteoclastogenesis independent of RANKL-RANK signaling [37]. IL-1 is a potent mediator of pathological bone destruction induced by estrogen deficiency or inflammation [7]. Disrupting the type I IL-1 receptor or IL-1 signaling can reverse bone loss induced by ovariectomy [38] or rheumatoid arthritis [39]. This study demonstrated the ability of acteoside to reduce production of inflammatory cytokines such as TNF-α, IL-1β, and IL-6 in macrophages. Acteoside is thought to inhibit inflammatory cytokine production by suppressing p38 kinase and ERK signaling, because activating ERK1/2, p38 MAPK, or both is required for lipopolysaccharide-induced production of these cytokines in macrophages [40], [41]. Luteolin, an inflammatory compound, had also been reported to suppress the production of inflammatory mediators by inhibiting p38 MAPK activation [42].

In this study, we also found that acteoside attenuated bone loss in ovariectomized mice, as evidenced by the restored maximum fracture force at the mid shaft of the right femur and the disappearance of osteoporotic cortical bone. Oral acteoside administration down-regulated the ovariectomized-induced increases in serum IL-1β and IL-6 levels, but not ALP. The increased serum levels of calcium, TRAP, and OC in OVX were also inhibited by oral treatment with acteoside, suggesting that acteoside attenuates alteration of biomarkers specific for bone formation as well as resorption. As osteoporosis is characterized by a reduced mass density and deteriorated trabecular bone microarchitecture, OVX-induced trabecular bone loss and morphometric parameter alteration were significantly inhibited by oral acteoside administration. These findings suggest that acteoside may be used as an anti-resorptive agent to treat osteoporosis by reversing imbalanced osteoclast activation. However, osteoblasts are the primary factor responsible for new bone formation. Thus an agent able to increase osteoblast proliferation or differentiation is needed to enhance bone formation [30]. In contrast, we found that acteoside did not affect osteoblast differentiation or mineralization in DAG-treated bone marrow cells. Taken together, our results suggest that acteoside has an anti-resorption effect but does not directly affect bone formation. More detailed experiments analyzing bone-specific parameters *in vivo* and *in vitro* are needed to clarify whether or not acteoside benefits osteoblastogenesis.

The present study highlights the inhibitory effect of acteoside on osteoclast differentiation and bone resorption by suppressing MAPKs and several transcriptional factors such as NF-κB, c-Fos, and NFATc1. The data suggest two possible mechanisms by which acteoside has these benefits. One possibility is that acteoside inhibits osteoclastogenesis due to its antioxidant potential. Numerous studies have demonstrated that receptor-mediated ROS production may serve as a downstream signaling mediator [43]–[45]. A few kinases and transcription factors are sensitive to the cellular redox state, which affects various cellular events. RANKL stimulates ROS production, which mediates RANKL-induced cellular responses for osteoclast differentiation [24]. Pretreatment with antioxidants, such as *N*-acetyl cysteine and glutathione, prevented RANKL-mediated ROS generation, indicating that antioxidants reduce bone loss by lowering RANKL-induced ROS production [24]. Consistent with these findings, the present study reveals that acteoside attenuates intracellular ROS produced in BMMs during osteoclast differentiation in a dose-dependent manner. This observation suggests that the inhibition of osteoclastogenesis is at least in part due to the antioxidant potential of acteoside. We also suggest that acteoside might down-regulate Ca^2+^ influx, thus suppressing osteoclastogenesis. Acteoside was recently reported to inhibit type I allergies by down-regulating Ca/NFAT and JNK signaling in basophilic cells [46]. The calcium-sensing receptor is closely related to the regulation of osteoclastogenesis [47]. This relationship suggests that a calcium channel is involved in acteoside-induced inhibition of osteoclast differentiation and formation. However, further studies are required to explore the exact mechanisms by which acteoside acts as an anti-resorptive agent through modulation of Ca^2+^ homeostasis.

In conclusion, our present findings show that acteoside inhibits RANKL-induced osteoclast differentiation from BMMs and RAW264.7 macrophages and suppresses bone resorption by mature osteoclasts. Acteoside also prevents RANKL-induced activation of three well-known MAPKs and transcription factors such as NF-κB, c-Fos, and NFATc1, as well as the production of inflammatory cytokines such as TNF-α, IL-1β, and IL-6. In addition, oral administration of acteoside attenuates ovariectomy-induced osteoporosis, though it does not affect osteoblastogenesis from bone marrow cells. Collectively, these findings suggest that acteoside has beneficial roles in reducing the osteoclast formation and activity as a potent anti-resorptive agent.

## Supporting Information

Figure S1 **Chemical structure of acteoside.**
(TIF)

Figure S2 **Acteoside prevents RANKL-induced pit formation in BMMs.** A, BMMs were pretreated with the indicated doses of acteoside for 2 h in bone-coated 24-well plates and stimulated with 50 ng/ml M-CSF and 100 ng/ml RANKL for 7 days. Pit formation was observed under optic microscopy. B, BMMs were cultured with M-CSF and RANKL in the presence of various acteoside concentrations (0–20 µM), and 7 days later, the resorbed area was quantified from 3 independent experiments and expressed as percentage of control (*n* = 4 per experiment). ^*^ *p*<0.05, ^**^ *p*<0.01, and ^***^ *p*<0.001 vs. cells cultured with M-CSF and RANKL.
(TIF)

Figure S3 **Acteoside attenuates the production of inflammatory cytokines in RANKL-stimulated RAW264.7 cells.** Cells were pretreated with the increasing concentrations (0–10 µM) of acteoside for 2 h followed by stimulation with 100 ng/ml RANKL for 48 h. The levels of TNF-α, IL-1β, and IL-6 were determined by using ELISA kits. ^***^ *p*<0.001 vs. cells without RANKL and acteoside. ^#^ *p*<0.05 and ^##^ *p*<0.01 vs. cells stimulated with RANKL only.
(TIF)
