# Supplementary figures and images for: Acteoside Suppresses RANKL-Mediated Osteoclastogenesis by Inhibiting c-Fos Induction and NF-κB Pathway and Attenuating ROS Production
Source: PLoS One. 2013 Dec 4;8(12):e80873. doi: 10.1371/journal.pone.0080873 (PMC3851776; doi:10.1371/journal.pone.0080873)

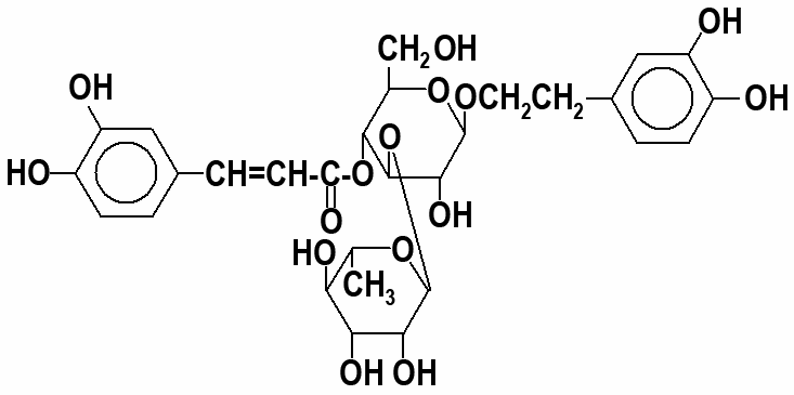

Supplement: Figure S1 — Chemical structure of acteoside. (TIF) [file pone.0080873.s001.tif]
